# 5-FU therapeutic drug monitoring as a valuable option to reduce toxicity in patients with gastrointestinal cancer

**DOI:** 10.18632/oncotarget.24338

**Published:** 2018-01-30

**Authors:** Katarzyna Morawska, Françoise Goirand, Laurine Marceau, Madeline Devaux, Adèle Cueff, Aurélie Bertaut, Julie Vincent, Leila Bengrine-Lefevre, François Ghiringhelli, Antonin Schmitt

**Affiliations:** ^1^ Centre Georges-François Leclerc, Dijon, France; ^2^ Laboratoire de Pharmacologie/Toxicologie, CHU de Dijon, Dijon, France; ^3^ INSERM U1231, University of Burgundy Franche-Comté, Dijon, France

**Keywords:** 5-FU, therapeutic drug monitoring, adverse event, GI cancer, pharmacokinetics

## Abstract

**Aims:**

5-FU is used as the main backbone of chemotherapy regimens for patients with colorectal and other gastrointestinal cancers. Despite development of new strategies that allowed enhancing clinical effectiveness and tolerability of 5-FU, 10–30% of patients treated with 5-FU-based regimens experience severe treatment-related toxicity. In our study, we evaluated the 5-FU exposure-toxicity relationship and investigated the efficacy of PK-guided dosing in increasing tolerability of 5-FU-based chemotherapy.

**Results:**

50.7% of patients required dose adjustments after cycle 1. Percentage of patients within 5-FU AUC range was 49.3%, 66.9%, 61.0% at cycle 1, 2 and 3 respectively (*p* = 0.002 cycle 1 vs cycle 2). At all 3 cycles, lower incidences of grade I/II toxicities were observed for patients below or within range compared with those above range (19.4% vs 41.3%, *p* < 0.001 respectively).

**Conclusions:**

Our analysis confirms that the use of BSA-guided dosing results in highly variable 5-FU exposure and strongly suggests that PK-guided dosing can improve tolerability of 5-FU based chemotherapy in patients with gastrointestinal cancers, thus supporting 5-FU therapeutic drug monitoring.

**Methods:**

155 patients with gastrointestinal cancers, who were to receive 5-FU-based regimens were included in our study. At cycle 1, the 5-FU dose was calculated using patient’s Body Surface Area (BSA) method. A blood sample was drawn on Day 2 to measure 5-FU concentration. At cycle 2, the 5-FU dose was adjusted using a PK-guided dosing strategy targeting a plasma AUC range of 18–28 mg·h/L, based on cycle 1 concentration. Assessments of toxicity was performed at the beginning of every cycle.

## INTRODUCTION

5-Fluorouracil (5-FU) is widely used in the treatment of solid malignancies, especially in colorectal and other gastrointestinal cancers, both in advanced and adjuvant settings [1]. Over the past few years, increased understanding of its mechanism of action has led to the development of new strategies, such as addition of the biochemical modulator leucovorin, combination with other cytotoxic agents or administration by continuous intravenous infusion, which allowed achieving higher tolerable doses (and thus higher exposures) and enhancing clinical effectiveness [2–4]. Despite these advances, 10%–30% of patients treated with fluoropyrimidines as monotherapy experience severe treatment-related toxicity leading to death in 0.5%–1% of the cases without DPD deficiency [5–7] and even higher when dosed with irinotecan and/or oxaliplatin [8]. Consequently, determination of biomarkers that could predict toxicity of chemotherapeutic agents, including 5-FU, remains a central goal of recent research in oncology [1].

5-FU dosing is traditionally determined according to body surface area (BSA). A substantial body of evidence demonstrates that BSA-based dosing is associated with wide intra-individual pharmacokinetic variability resulting in significant differences in 5-FU exposure. Therefore, identical doses of 5-FU in different patients often result in different drug exposure leading to under- or overexposure in many patients [9–17]. 5-FU along with many other cytotoxic drugs is characterized by a strong toxicity-exposure relationship and narrow therapeutic window, which support the use of therapeutic drug monitoring (TDM) approaches [3, 18, 19]. Efficacy of 5-FU TDM has been validated in two multicentric randomized trials, which demonstrated significant superiority of pharmacokinetically-guided dosing (PK-guided dosing) compared with BSA-based dosing to decrease grade III/IV toxicity and improve objective response rate [13, 14]. Area Under the Curve of 5-FU concentrations versus time (AUC) is considered as the most relevant pharmacokinetic parameter associated with 5-FU-related efficacy and toxicity. Some authors have proposed target AUC when 5-FU is dosed as a bolus during 5 days every 4 weeks [20–22]. However, because of the very short 5-FU half-life, several blood samples in a relatively limited amount of time are needed. Since 5-FU, in colorectal cancer, is now mostly administered by continuous intravenous infusion over several days, AUC is easily determined based on steady state plasma concentration [18]. Several previous studies proposed a 5-FU target AUC of 20–24 mg.h/L [13, 16]. However, because of its intrinsic variability, it is tough to remain in such a narrow therapeutic window, and thus, it is generally considered that AUC range of 20–30 mg.h/L is required for successful therapy [14, 18]. In our center, we have decided to use a 18–28 mg.h/L target AUC, based on Gamelin’s algorithm (Table 1). In Gamelin’s paper, the target AUC was 20–24 mg.h/L. Dose adaptation of +5% or −5% were required for patients with AUC comprised between respectively 18–20 mg.h/L and 24–28 mg.h/L. Because of the precision of 5-FU measurements, such a small dose modification would not have a clinical nor biological incidence. Thus, we increased the target AUC to start dose adjustment at ±10%.

**Table 1:** 5-FU dose adaptation algorithm used in the present study and the one proposed by Gamelin

AUC (mg.h/L )	5-FU Dose Adjustment (± % of previous dose)	Gamelin’s algorithm
<4	+70	+70
4 to < 8	+50	+50
8 to < 10	+40	+40
10 to <12	+30	+30
12 to < 15	+20	+20
15 to < 18	+10	+10
**18 to < 20**	**Unchanged**	+5
**20 to < 24**	**Unchanged**	**Unchanged**
**24 to < 28**	**Unchanged**	−5
28 to < 31	−10	−10
>31	−15	−15

5-FU PK variability is affected by various factors such as age, gender, disease status, organ functions, drug-drug interactions, however the most well-known cause of 5-FU intolerance is deficiency of dihydropyrimidine dehydrogenase (DPD) activity - the key enzyme responsible for its metabolism [10]. DPD deficiency is observed in 39–61% of patients developing severe toxicity [6]. As a result, polymorphisms in *DPYD*, the gene encoding DPD, have gained widespread attention as predictors of fluoropyrimidines-related toxicity. More than 30 sequence variations in the *DPYD* gene have been yet identified, while the most well-established variant is DPYD*2A [3]. Clinical Pharmacogenetics Implementation Consortium has established the fluoropyrimidines dosage algorithm based on the interpretation of clinical *DPYD* genotype tests. Initial dose reduction of at least 50% is proposed for patients heterozygous for DPYD*2A, DPYD*13 and c.2846A > T, who are considered to have intermediate or partial DPD enzyme activity, while a choice of alternative drug is strongly recommended for patients with complete DPD deficiency [23]. However, DPD activity is regulated not only at the level of *DPYD* gene, but at the transcriptional and the post-transcriptional levels as well [7]. It highlights the significant limitation of the proposed algorithm. For this reason, other strategies assessing DPD activity, such as DPD phenotyping are investigated [7, 24–26].

DPD converts uracil (U), its endogenous substrate, into dihydrouracil (DHU), and the pretreatment DHU/U ratio or uracil concentrations alone have the great potential to identify patients at risk of severe fluoropyrimidine-associated toxicity [7, 27]. According to certain studies, the DHU/U ratio correlates with clearance of 5-FU and risk of its toxicity, however despite strong evidence on its clinical validity, the use of the DHU/U ratio in daily clinical practice is still limited [24–26]. Moreover, the fact that approximately 50% of patients who experience 5-FU toxicity will have no documented alterations in the DPD activity, suggests that DPD genotyping or phenotyping should be performed in combination with a more clinically relevant parameter such as 5-FU plasma level [3].

Given the evidence for 5-FU TDM efficacy, we conducted this prospective study to investigate the value of PK-guided 5-FU dosing in decreasing toxicity in 155 patients with gastrointestinal cancer receiving 5-FU-based regimens such as simplified FOLFOX-6, FOLFIRINOX, FOLFIRI, FOLFIRI-3 and LV5FU2. The primary objective was to demonstrate the ability to achieve a target 5 FU AUC of 18–28 mg.h/L within 3 cycles of chemotherapy. Secondary objectives were to assess the 5-FU exposure-toxicity relationship, the ability to decrease 5-FU related toxicity during subsequent cycles and the impact on efficacy in a small and homogenous group of patients.

## RESULTS

### Patient population and treatment

A total of 155 patients (66 females and 89 males) were included in our study. Their mean age was 66 ± 10.6 (range, 27–87) years. The primary tumor sites were colon/rectum (60.0%), pancreas (21.3%), esophagus (7.0%), stomach (6.5%) and others (5.2%). 5-FU was administered as an intravenous bolus followed by a 46-h continuous infusion (2400 mg/m^2^ initially, then adjusted) through a portable infusion pump starting on day 1 of every cycle. Patients were treated with routinely used regimens such as simplified FOLFOX-6 (39.4%), FOLFIRINOX (32.3%), FOLFIRI (18.1%), LV5FU2 (5.7%) and FOLFIRI-3 (4.5%) with or without concomitant biological therapy. Subsequent cycles were repeated every 2 weeks. One hundred and fifty-five patients received a first cycle of chemotherapy, 154 received a second cycle and 118 only received a third cycle (37 patients discontinued the therapy due to toxicity development, disease progression, surgical intervention or treatment pause). Patient’s characteristics are listed in Table 2.

**Table 2:** Baseline patient characteristics

Characteristics	*n*
**No. of evaluable patients**	155
**Gender**	
Male	89 (57.4%)
Female	66 (42.6%)
**Age (range)**, years	65.8 ± 10.64 (27–87)
**Weight (range)**, kg	69.7 ± 14.76 (35–115)
**Height (range)**, cm	168.1 ± 8.90 (131–186)
**Location of cancer**	
Colorectal	93 (60.0%)
Pancreas	33 (21.3%)
Esophagus	11 (7.0%)
Stomach	10 (6.5%)
Others	8 (5.2%)
**Type of chemotherapy**	
Metastatic	128 (82.6%)
Adjuvant	27 (17.4%)
Protocol of chemotherapy	
Simplified Folfox-6	61 (39.4%)
Folfirinox	50 (32.3%)
Folfiri	28 (18.1%)
LV5FU2	9 (5.7%)
Folfiri-3	7 (4.5%)
**Biotherapy**	
Yes	74 (47.7%)
No	81 (52.3%)
**Line of treatment**	
1st line	80 (51.6%)
2nd line	45 (29.0%)
3rd line or more	30 (19.4%)

### Intra-individual variability of 5-FU pharmacokinetics

**Figure 1:**
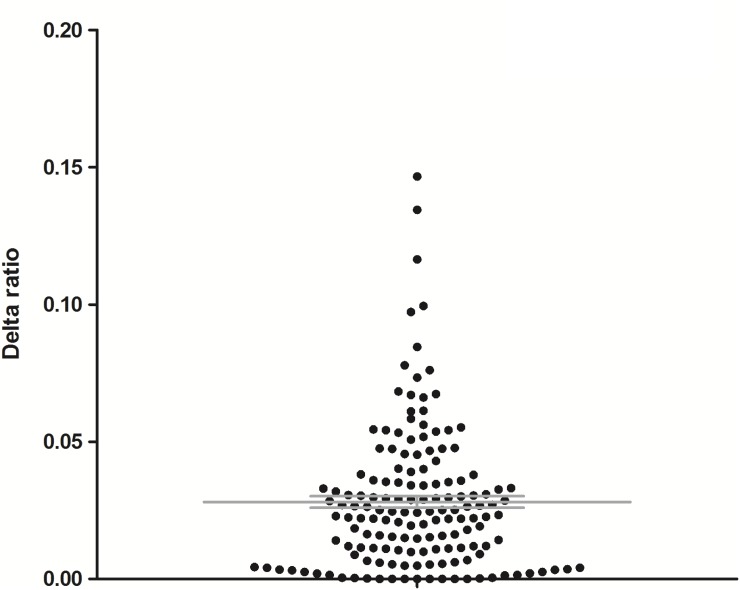
Differences between the highest and lowest concentration/dose ratios among 3 cycles for each patient (delta ratios)

As shown in Figure 1, for the majority of patients (83%) the values of delta ratios (i.e., difference between the highest and the lowest 5-FU AUC/dose ratio for a patient) were smaller than 0.05 (resulting in mean value of 0.028 ± 0.026), which means that even in case of dose adaptation the ratio remained relatively stable, thus the intra-individual variability is low. We also calculated the intra-individual variability for the ratio which resulted in a mean coefficient of variation of 17%.

### Inter-individual variability in 5-FU pharmacokinetics

**Figure 2:**
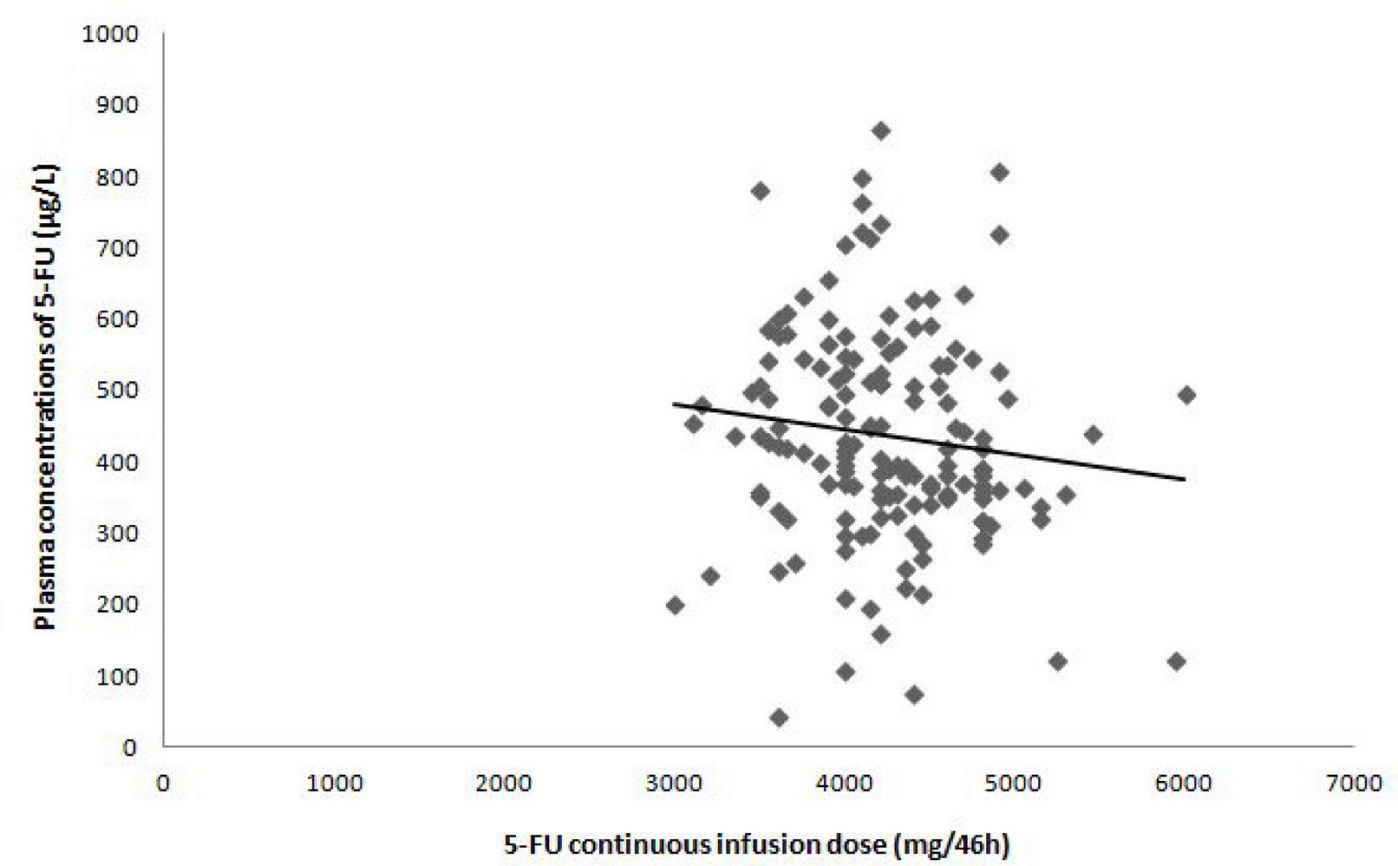
Relationship between 5-FU plasma concentration and 5-FU continuous infusion dose at cycle 1

At cycle 1, the 5-FU concentrations were plotted against the 5-FU 46 h-infusion dose. As shown in Figure 2, patients who received high doses were often the one with relatively low concentrations. Moreover, considerable differences in blood concentrations were observed for the same 5-FU doses (e.g., for a total dose of 4000 mg/46 h, steady-state concentrations were ranging from 110 μg/L to 706 μg/L) underlining an important inter-individual variability of 5-FU pharmacokinetics (*R*^2^ = 0.01448) and consequently the need of dose individualization.

### Impact of 5-FU PK-guided dosing adjustment to reach target AUC

**Figure 3:**
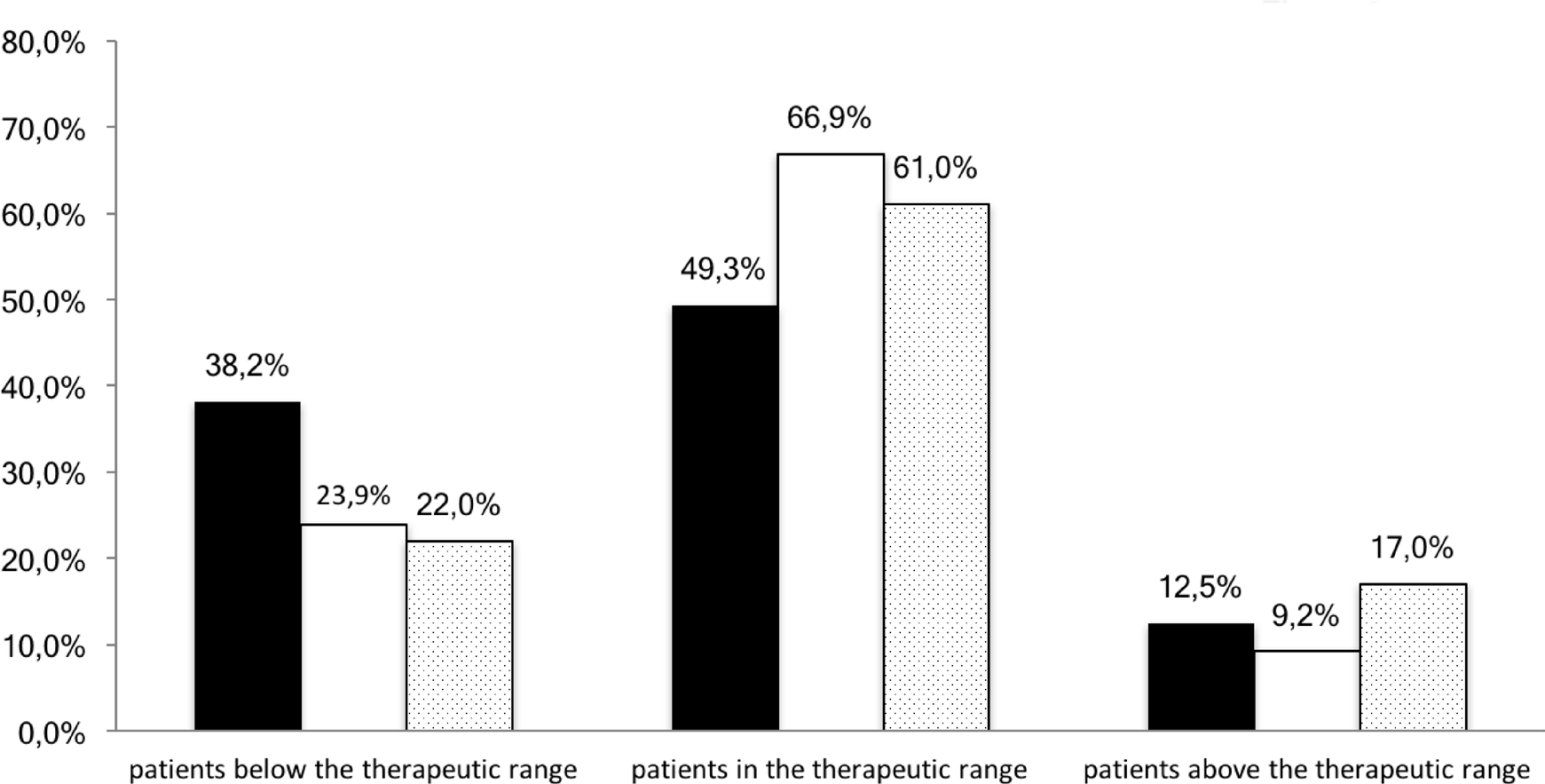
Percentage of patients with 5-FU AUC values below, within or above the therapeutic range for each cycle (black columns: cycle 1, white columns: cycle 2, dotted columns: cycle 3)

The next step of the present study was to assess if PK-guided dosing adjustment between the cycle 1 and 2 increased the percentage of patients in the therapeutic range. PK-guided dose adjustment after cycle 1 was required for 50.7% of patients (77 of 152). Percentage of patients within range significantly increased from 49.3% at cycle 1 (75 of 152) to 66.9% at cycle 2 (95 out of 142) (*p* = 0.002). At cycle 3, the number of patients in the target AUC non-significantly decreased to 61.0% (*p* = 0.371). PK-guided 5-FU dosing tended to result in less underexposed patients (i.e. <18 mg.h/L ) at cycle 2 (23.9%, 34 of 142, *p* = 0.009) and cycle 3 (22.0%, 18 of 82, *p* = 0.066) than at cycle 1 (38.2%, 58 of 152) (Figure 3).

At cycle 1, higher mean AUC values were observed for women (21.7± 6.2 mg.h/L ) than for men (18.7 ± 7.2 mg.h/L ) (*p* < 0.001) while the mean doses administered to female patients were significantly lower as compared with male (3993 ± 455 mg and 4426 ± 478 mg, *p* < 0.001, respectively).

The mean AUC increased from 20.0 ± 6.9 mg.h/L at cycle 1 to 21.8 ± 6.3 mg.h/L at cycle 3 and AUCs overall variability tend to decrease as reflected by its coefficient of variation (34.6% at cycle 1 and 29.0% at cycle 3).

### Relation between 5-FU exposure and toxicity

**Figure 4:**
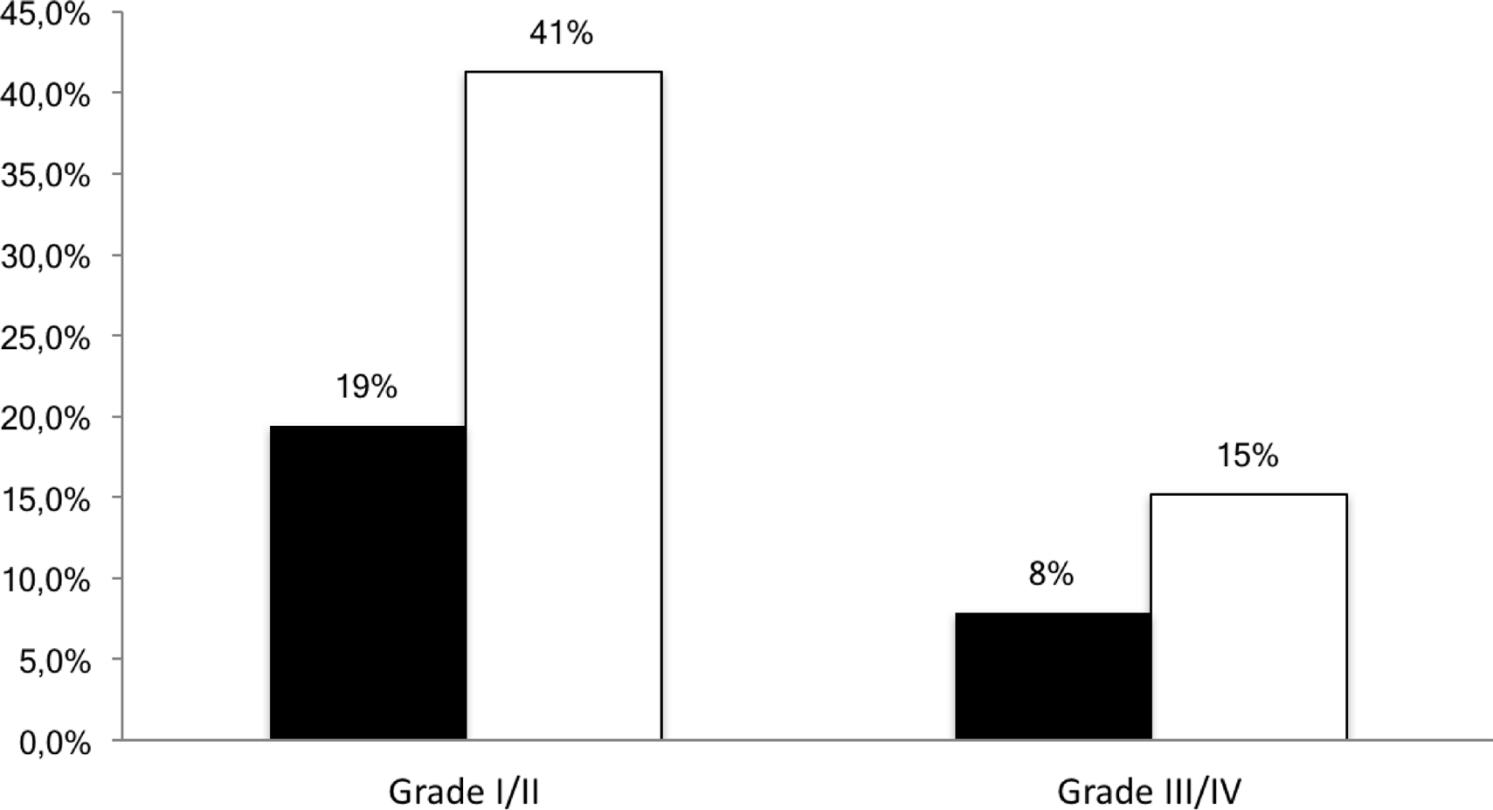
Percentage of patients developing grade I/II or III/IV toxicity according to the AUC range (black columns: below or within the therapeutic range, white columns: above the therapeutic range)

At all 3 cycles, we observed significantly lower incidences of grade I/II toxicities for patients below or within range (19.4%) compared with those above range (41.3%) (*p* < 0.001). For grade III or IV toxicities, percentage of patients below or within range (7.9%) compared with those above range (15.2%) did not differ significantly (Figure 4).

**Figure 5:**
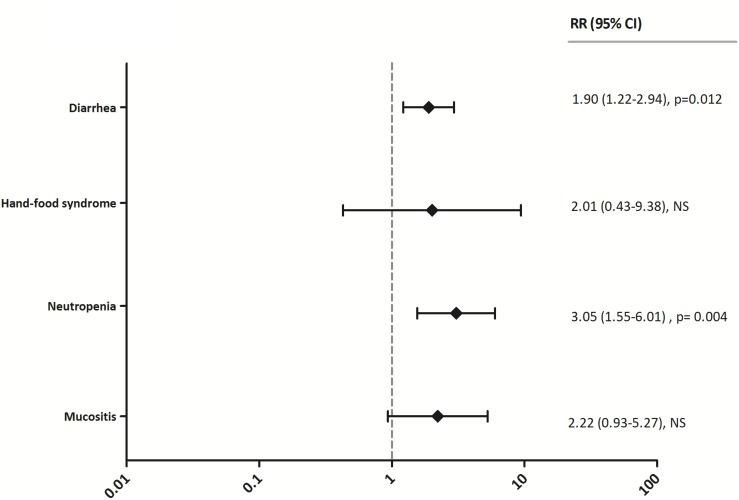
Relative risks of grade I/II and III/IV diarrhea, hand-food syndrome, neutropenia and mucositis in patients below or within the AUC range versus overexposed patients RR > 1 reflects higher incidence in the overexposed group, while RR < 1 represents higher incidence in patients below or within the AUC target. RR = Relative Risk, CI = Confidence Interval, NS = Not Significant, I/II = grade I/II, III/IV = grade III/IV.

Relative risk was also determined for adverse effects evaluated in this study (Figure 5). All grades of neutropenia were more frequent in overexposed patients than in patients below or within the AUC range (RR: 3.05; 95% CI: 1.55–6.01, *p* = 0.004). Significantly higher incidences of diarrhea (RR: 1.90; 95% CI: 1.22–2.94, *p* = 0.012) were observed. The frequency of hand-food syndrome and mucositis was also more common in the group above therapeutic range (RR: 2.01; 95% CI 0.43–9.38, NS and RR: 2.22; 95% CI 0.93–5.27, NS, respectively), however the differences were not statistically significant.

### Toxicity incidence at subsequent cycles

As indicated in Table 3, the incidence of all grades of adverse events remained constant or decreased during subsequent cycles. Increase in the incidence of grade I/II mucositis was also noted at cycle 2 (6.5%) compared with cycle 1 (5.2%). However, none of the presented results were statistically significant.

**Table 3:** Frequency of evaluated toxicities at subsequent cycles

	Cycle 1 (*n* = 155)			Cycle 2 (*n* = 154)			Cycle 3 (*n* = 118)		
Toxicity	All grades	Grade I/II	Grade III/IV	All grades	Grade I/II	Grade III/IV	All grades	Grade I/II	Grade III/IV
**Diarrhea**	22.6% (35)	19.4% (30)	3.2% (5)	20.1% (31)	16.9% (26)	3.2% (5)	12.7% (15)	11.9% (14)	0.8% (1)
**Hand-food syndrome**	3.2% (5)	3.2% (5)	0% (0)	2.6% (4)	2.6% (4)	0% (0)	0% (0)	0% (0)	0% (0)
**Neutropenia**	9.7% (15)	3.9% (6)	5.8% (9)	6.5% (10)	1.3% (2)	5.2% (8)	7.3% (9)	5.1% (6)	2.5% (3)
**Mucositis**	8.4% (13)	5.2% (8)	3.2% (5)	6.5% (10)	6.5% (10)	0% (0)	2.5% (3)	2.5% (3)	0% (0)

Only 10 patients (6.5%) benefited from 5-FU bolus dose reduction between cycle 1 and cycle 2, due to > grade 2 toxicity. Out of those 10 patients, 8 were below or within the target AUC and only 2 were above. Those 2 last patients have also benefited from an infusion dose reduction.

Importantly, we performed two-paired signed rank test comparing the toxicity grades at cycle 1 and 3 for each patient (*n* = 118), which resulted in a significant decrease in toxicity at cycle 3 compared with cycle 1 (*p* = 0.018). This decrease in toxicity was associated, for patients who developed III/IV toxicities at cycle 1, with a dose decrease from 4122.2 mg ± 520.5 at cycle 1 to 4025.0 mg ± 570.9 and 3921.4 mg ± 796.8 at subsequent cycle 2 and 3.

Lastly, a significantly higher number of women experienced any grade toxicities at cycle 1 as compared with men (43.9% vs 22.5%, *p* = 0.004).

### Efficacy assessment

**Figure 6:**
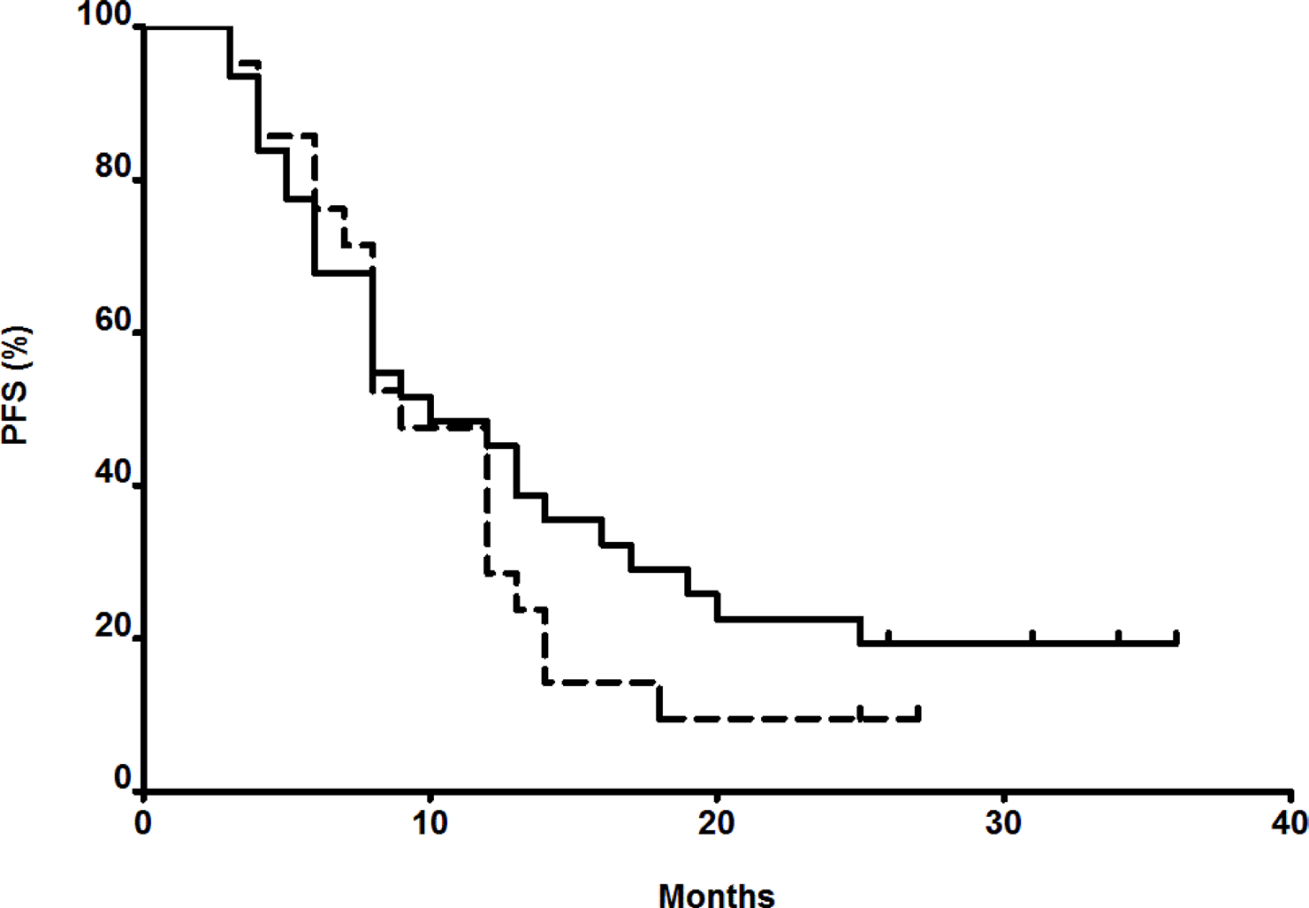
Progression-Free Survival (PFS) of metastatic colon cancer patients in first or second line Dash line: patients with at least one AUC below the target during the 3 first cycles. Black line: patients always within or higher than target during the 3 first cycles.

Progression-Free Survival (PFS) was assessed in an homogenous subset of patients: 52 metastatic colon cancer patients in first or second line. Median PFS for patients at least once below the target AUC (*n* = 21) versus patients always within or above the target (*n* = 31) were not statistically different (i.e., PFS of 9 and 10 months respectively). In the group of patients always within or above the target AUC, 25.8% of them had a PFS of more than 20 months, while they were only 9.5% in the other group (Figure 6).

## DISCUSSION

5-FU has been a leading drug in PK-guided cancer chemotherapy due to the strong exposure-toxicity relationship and an important inter-individual variability in its pharmacokinetics. Thus, a number of studies have been conducted in order to evaluate an appropriate dose adjustment algorithm and reflect the advantage of 5-FU PK-guided dosing to reduce toxicity and enhance therapeutic outcomes [10, 11, 13–16]. Taking into account that the standard guidelines for the treatment of gastrointestinal cancer has shifted toward combination therapy, the aim of the present study was to extend this type of investigation in chemotherapy regimens such as simplified FOLFOX-6, FOLFIRINOX, FOLFIRI, FOLFIRI-3 and LV5FU2 and evaluate the efficacy of PK-guided dose adjustment in daily clinical practice in terms of toxicity management and efficacy. In this study, an optimal AUC range of 18–28 mg.h/L was suggested and the dose adaptation algorithm was based on analysis presented in current literature data [12, 13].

More than half of all patients included in our study (50.7%) required dose adaptation after cycle 1 (BSA-based dosing). Of these, 38.2% required an increase in dose and 12.5% a dose reduction in order to achieve the AUC within the range. PK-guided 5-FU dosing performed at cycle 2 resulted in significantly more patients achieving the target AUC (66.9%). Dose adaptation in case of under- or over-exposure was not mandatory in our study. Consequently, some oncologists have sometimes, for clinical reasons, decided not to follow the dose-adaptation recommendation. This explains why only about 67% of patients are in the therapeutic range at cycle 2. The study of Patel *et al.* [16] which included 70 colorectal cancer patients receiving mFOLFOX-6 regimen resulted in 29.6% of patients in the therapeutic range, 51.9% of patients who were under-dosed and 18.5% of patients above the range. Gamelin *et al.* [13] compared standard dosing with therapeutic dose monitoring in 208 patients treated with 5-FU-based regimen. 17.3% patients in PK-guided group, who received their first BSA-based dose, were above the therapeutic level, 14.4% were in therapeutic range and 68% were underexposed. Results presented in mentioned studies and our investigation demonstrate that the vast majority of patients is not in the expected therapeutic range after receiving standard 5-FU BSA-based dose. We can therefore assume that BSA-based 5-FU dosage is associated with significant pharmacokinetic variability and may be of limited interest. This variability appeared to be smaller during cycle 2 and 3, after performing 5-FU dose adjustment.

As previously described, the PK-guided dose adjustment algorithm can also reduce the risk of toxicity by keeping individual AUC out of the toxic range [2, 3, 9, 28]. The 5-FU-related toxicities evaluated in our study at all 3 cycles were associated with the drug exposure. Significantly lower incidences of grade I/II toxicity were observed for patients below or within range (19.4%) compared with those above range (41.3%). For grade III or IV toxicity the differences of 7.9% versus 15.2% were reported, however probably due to low incidence rate of this high-grade toxicities, they were not significant. The relative risk of any toxicity grade was greater for all evaluated adverse effects, however the difference for hand-food syndrome and mucositis was not statistically significant. Patel *et al.* [16] in 70 colorectal patients receiving mFOLFOX6 regimen observed significantly higher rates of any grade III/IV toxicity (e.g. neutropenia diarrhea, mucositis, nausea, fatigue) in patients above the therapeutics range compared with those below or within AUC threshold. Specifically, the incidence of III/IV neutropenia and diarrhea was higher in over-exposed patients, however mucositis appeared to be slightly more frequent in patients within range. This could be explained by small number of patients developing grade III/IV mucositis in present and Patel’s investigation (5 out of 154 and 1 of 54 at all 3 cycles, respectively). Moreover, although previous studies [29, 30] reported a strong correlation between hematological toxicity, diarrhea and mucositis, the last one appeared to be more influenced by 5-FU bolus rather than continuous infusion dosage [4]. In addition, our study included patients treated by various combination therapy regimens with components that could influence the incidences of toxicity in performed analysis.

In order to determine the utility of dose adjustment algorithm in daily clinical practice, we evaluated 5-FU related toxicities during subsequent cycles. A significant decrease in toxicity at cycle 3 compared with cycle 1 was observed. For the same purpose, efficacy was assessed only in metastatic colon cancer patients receiving a 1st or 2nd line of chemotherapy, in order to have comparable PFS. Patients within or above the target AUC during the first 3 cycles were compared to the one with a least one AUC below. Despite the lack of difference in median PFS, there is a trend to a shortened PFS for patients with at least one AUC below the target. A significant body of studies demonstrated better tolerability and superior efficacy of 5-FU in PK-guided dose adjustment arm in contrast with BSA-based dosage arm [11, 13, 14]. The single-arm design is a principal limitation of our study, however it allowed to evaluate PK-guided 5-FU dose adjustment in daily clinical oncology practice.

The main limitation of this work is that, at the time of the study, DPD genotyping or phenotyping were not available in our institution. Thus, dose adaptation at cycle 1 based on those criteria was not manageable. However, we believe that there is a real interest in detecting, as early as the first cycle, patients at risk. Consequently, a DPD evaluation in order to select a non-toxic dose at cycle 1, and then dose adaptation on further cycles, based on exposure, would be the best solution in order to avoid toxicity and increase efficacy. For example, as proposed by Launay *et al.* [31], a pre-therapeutic screening of DPD activity by phenotyping could be proposed. Patients with no DPD deficiency would receive full dose, and patients with mild, intermediate or profound deficiency would see their doses decrease by respectively 15%, 30% or 50%. Then, depending on the observed 5-FU concentrations and observed toxicity, dose would be increased or decreased accordingly.

Another point that needs to be taken in account is linked to the well-known important within-day variability of 5-FU [32, 33]. In order to limit this variability, in our institution, blood samples are drawn always at the same time (i.e., on day 2, between 8 and 10 am). With such a procedure, variability between 2 cycles was not higher than 17%, allowing the prediction of cycle 2 5-FU exposure based on cycle 1 data.

As early as 2001, due to the absence of rationale to BSA-dosing for most cytotoxic and in order to simplify drug preparation, some authors have proposed to round dose (i.e., dose-banding) chemotherapy [34]. This approach is already applied in several institutions for some drugs [35, 36]. However, due to this very easily-applicable TDM, it seems that 5-FU is probably not the best drug candidate for such an approach.

At cycle 1, mean AUC values observed for women were significantly higher as compared with men, despite the same initial dose of 2400 mg/m^2^ and, due to smaller BSA in women, lower 5-FU total dose. Additionally, we found greater incidence of all grade toxicities in female patients as already demonstrated in earlier studies [37–40]. A partial explanation of these differences may be a decreased clearance of 5-FU among women related to the DPD activity, which was reported to be lower in women as compared with men [41]. This explanation is confirmed by the fact that, at cycle 2, after dose adaptation, AUC and toxicity are identical between men and women. Thus, this higher risk of toxicity for women is more linked to over-exposure than over-sensitivity to 5-FU toxic effect. This gender-related exposure leading to 5-FU-related toxicity raises an important question whether the initial dose of 5-FU for female patients should be lower than that for men.

## PATIENTS AND METHODS

### Patients

All patients with gastrointestinal cancer who were to receive a 46-h continuous 5-fluorouracil infusion based protocol from April 2014 to February 2016 in Dijon’s Clinical Cancer Center were included. All patients routinely underwent a blood analysis in order to evaluate their 5-FU exposure during the 3 first cycles. Consequently, no informed consent was required. However, data used in this manuscript were recorded in such a manner that subjects could not be identified. Patient confidentiality was maintained and the protocol for data collection and analysis was in compliance with guidelines and approved by our Institutional Review Board.

### 5-FU administration, blood sampling and plasma concentration determination

5-FU was administered continuously through a portable pump (BodyGuard 323 Colorvison, CME, Israel), allowing a controlled flow rate over the entire 46-h. Blood samples were drawn the day following the beginning of a 46-h continuous infusion (between 8 am and 10 am) during the 3 first cycles. Samples were immediately centrifuged and plasma kept frozen at −20°C until analyzed. Plasma 5-FU concentrations were determined by liquid chromatography as described previously [33]. Chloro-uracil was used as internal standard. 5-FU was extracted from the plasma with isopropanol-ethyl acetate (15/85 v:v) in the presence of 200 mg ammonium sulfate to precipitate proteins. The organic phase was dried at 50°C under nitrogen dioxide and reconstituted with 200 μL mobile phase before injection. Mobile phase consisted of methanol/water (5/95 v:v). *UV* detection was performed at 265 nm. This method was fully validated for routine measurement of 5-FU with a lower limit of quantification of 30 μg/L. The linearity was assessed from 30 μg/L to 2000 μg/L. Interday variations of the method was evaluated using two levels of QC samples (250 and 500 ng/mL). Interday precision was 10.9% and 7.9% (*n* = 9) respectively, for the two levels tested.

### Determination of intra-individual variability in 5-FU pharmacokinetics

In order to confirm the linearity of 5-FU pharmacokinetics within a patient, over cycle, we calculated ratios between 5-FU plasma concentration and its dose for all patients and for each cycle. Then, the delta ratio, defined as the difference between the highest and the lowest values among 3 findings, was calculated. A low difference represents low intra-individual variability.

### Inter-individual variability in 5-FU pharmacokinetics

Area Under the Curve of 5-FU concentrations *vs.* time (AUC) values, representing 5-FU exposure, were calculated by multiplying the 5-FU steady state concentration (mg/L) by the total infusion time (i.e., 46 h). During cycle 1, the 5-FU continuous infusion dose was based on patient body surface area (BSA-based dosing). Beginning with cycle 2, the 5-FU continuous infusion dose was then adapted according to an algorithm, derived from Gamelin’s one [13], based on the results of the AUC values from the previous cycle, targeting an AUC range of 18–28 mg.h/L (PK-guided dosing). No dose adaptation was performed after the fourth cycle, except development of 5-FU-related toxicity where doses were reduced. Clinicians were free to individually adapt 5-FU bolus or any other drugs doses included in the protocol.

### Toxicity assessment

Assessments of diarrhea, neutropenia, mucositis and hand-and-foot syndrome were performed at the beginning of every cycle. All adverse effects were clinically or biologically evaluated and graded according to the National Cancer Institute Common Terminology Criteria for Adverse Events version 4.0.

### Data analysis

In the present study, the primary objective was to demonstrate that 5-FU PK-guided dosing (i.e., for cycle 2 and 3) improves the ability to achieve an optimal target AUC (i.e. 18–28 mg.h/L). Percentage of patients within range at 3 cycles was compared using χ^2^ test. The mean AUC values for female and male patients were compared using U-Mann-Whitney test.

The secondary objectives were to demonstrate lower frequency of 5-FU-related toxicity after PK-guided dosing and assessment of efficacy. Relative risks of adverse effects incidence for patients above or within range compared with those who were overexposed were analyzed with one-tailed Fisher’s exact test. Efficacy was assessed by comparing Progression-Free Survival (PFS) in an homogenous subset of patients with metastatic colon cancer and receiving 5-FU-based regimens in first or second line. PFS was calculated in months from the time of 5-FU-based chemotherapy initiation to date of documented progression or last follow-up. The log-rank test was used to assess statistical differences among 2 groups: patients with at least one AUC below the target during the 3 first cycles vs. patients always within or higher than the target during the 3 first cycles. In the present study, a *p*-value < 0.05 was considered as statistically significant.

## CONCLUSIONS

Our analysis confirms that the use of BSA-guided dosing results in highly variable 5-FU exposure and strongly suggests that PK-guided dosing can improve tolerability of 5-FU based chemotherapy in patients with gastrointestinal cancers. Despite some barriers to the effective implementation of 5-FU TDM, including sampling time, PK-guided dosing can be successfully performed in daily clinical practice using a simple methodology. As others phenotypic markers, such as plasma uracil concentrations, have been recently shown to be good predictors of fluoropyrimidine-associated toxicity [7], it is likely that its use in combination with 5-FU TDM will further improve the safety of patients treated with 5-FU.

## Abbreviations

5-FU: 5-FluoroUracil
AUC: Area Under the Curve of concentration vs. time
BSA: Body Surface Area
CI: Confidence Interval
DHU: DiHydroUracil
DPD: Dihydro-Pyrimidine Deshydrogenase
*DPYD*: Gene coding for DPD
PFS: Progression-Free Survival
PK: PharmacoKinetic
RR: Relative Risk
TDM: Therapeutic Drug Monitoring
U: Uracil
